# Botanically-Derived Δ^9^-Tetrahydrocannabinol and Cannabidiol, and Their 1:1 Combination, Modulate Toll-like Receptor 3 and 4 Signalling in Immune Cells from People with Multiple Sclerosis

**DOI:** 10.3390/molecules27061763

**Published:** 2022-03-08

**Authors:** John-Mark Fitzpatrick, Becky Hackett, Lisa Costelloe, William Hind, Eric J. Downer

**Affiliations:** 1 Discipline of Physiology, School of Medicine, Trinity Biomedical Sciences Institute, Trinity College Dublin, University of Dublin, D02 R590 Dublin, Ireland; fitzpaj1@tcd.ie (J.-M.F.); hackettb@tcd.ie (B.H.); 2 Department of Neurology, Beaumont Hospital, D09 V2N0 Dublin, Ireland; lisacostelloe@beaumont.ie; 3 GW Research Ltd., Sovereign House, Vision Park, Histon CB24 9BZ, UK; Will.Hind@jazzpharma.com

**Keywords:** multiple sclerosis, TLRs, innate immunity, cannabinoids, PBMC, inflammation

## Abstract

The innate immune response to bacterial and viral molecules involves the coordinated production of cytokines, chemokines, and type I interferons (IFNs), which is orchestrated by toll-like receptors (TLRs). TLRs, and their intracellular signalling intermediates, are closely associated with multiple sclerosis (MS) pathogenesis. Recent data from our laboratory reported that the plant-derived cannabinoids, Δ^9^-tetrahydrocannabinol (THC) and cannabidiol (CBD), regulate viral and bacterial inflammatory signalling pathways controlled by TLR3 and TLR4 in macrophages. The aim of this study was to assess the impact of THC and CBD, when delivered in isolation and in combination (1:1), on TLR3- and TLR4-dependent signalling in peripheral blood mononuclear cells (PBMCs) from people with MS (pwMS; *n* = 21) and healthy controls (HCs; *n* = 26). We employed the use of poly(I:C) and lipopolysaccharide (LPS) to induce viral TLR3 and bacterial TLR4 signalling, and PBMCs were pre-exposed to plant-derived highly purified THC (10 μM), CBD (10 μM), or a combination of both phytocannabinoids (1:1 ratio, 10:10 μM), prior to LPS/poly(I:C) exposure. TLR3 stimulation promoted the protein expression of the chemokine CXCL10 and the type I IFN-β in PBMCs from both cohorts. THC and CBD (delivered in 1:1 combination at 10 μM) attenuated TLR3-induced CXCL10 and IFN-β protein expression in PBMCs from pwMS and HCs, and this effect was not seen consistently when THC and CBD were delivered alone. In terms of LPS, TLR4 activation promoted TNF-α expression in PBMCs from both cohorts, and, interestingly, CBD when delivered alone at 10 μM, and in combination with THC (in 1:1 combination at 10 μM), exacerbated TLR4-induced TNF-α protein expression in PBMCs from pwMS and HCs. THC and CBD displayed no evidence of toxicity in primary PBMCs. No significant alteration in the relative expression of *TLR3* and *TLR4* mRNA, or components of the endocannabinoid system, including the cannabinoid receptor CB_1_ (encoded by *CNR1* gene) and CB_2_ (encoded by *CNR2* gene), and endocannabinoid metabolising enzymes, fatty acid amide hydrolase (*FAAH*) and monoacylglycerol lipase (*MGLL*), was determined in PBMCs from pwMS versus HCs. Given their role in inflammation, TLRs are clinical targets, and data herein identify CBD and THC as TLR3 and TLR4 modulating drugs in primary immune cells in vitro. This offers insight on the cellular target(s) of phytocannabinoids in targeting inflammation in the context of MS.

## 1. Introduction

The innate immune system is a conserved system of defence that discriminates between pathogens and self via intricate cellular responses governed by toll-like receptors (TLRs), a member of the family of signalling pattern recognition receptors [1]. TLRs are expressed in immune cells and cells of the nervous system [2] where they recognise pathogen-associated molecular patterns that include viral and bacterial molecules such as double-stranded RNA and lipopolysaccharide (LPS). Upon binding to the receptor, viral and bacterial molecules induce intracellular signalling via interferon (IFN) regulatory factors (IRFs), nuclear factor (NF)-κB and mitogen-activated protein (MAP) kinases, to coordinate the cellular production of cytokines, chemokines and type I IFNs. Such TLR signalling cascades are activated via a suite of adaptor proteins, most commonly myeloid differentiation factor 88 (MyD88) [3]. There are 10 functional TLRs expressed in humans, and all TLRs signal via the MyD88 adaptor, with TLR3 being an exception, signalling via Toll-Interleukin-1 Receptor (TIR)-domain-containing adaptor-inducing IFN-β (TRIF) adaptor to induce MyD88-independent signalling [4].

Neuroinflammation in autoimmune conditions is associated with dysregulation or over-activation of TLRs and, in the field of multiple sclerosis (MS), a body of literature indicates that TLRs, their adaptors, and signalling intermediates, are linked to the progression and/or etiology of MS [5,6,7,8]. Indeed, genetic knockout studies using experimental autoimmune encephalomyelitis (EAE) indicate that TLR4 [9], TRIF [10], and IFN-β [11] deficiency exacerbate EAE, while knockout of MyD88 [9,12,13] and IRF3 [14] is protective in EAE, indicating the complex role of TLR pathways in inflammatory changes associated with mouse models of MS. In terms of human studies, the expression profile of TLR3 and TLR4 is elevated in active MS brain lesions [15], and TLR4 is upregulated in CSF mononuclear cells from people with MS (pwMS) [16], with data from our laboratory demonstrating that immune cells from pwMS are hypersensitive to LPS in terms of pro-inflammatory cytokine expression [17]. In addition, peripheral blood mononuclear cells (PBMCs) from pwMS, when compared to PBMCs from control subjects, are refractory to TLR3 stimulation, in terms of IFN-β production [18], indicating that TLR signalling may shape inflammatory responses to viral and bacterial infection at a cellular level in pwMS.

Cannabidiol (CBD) and Δ^9^-tetrahydrocannabinol (THC) are phytocannabinoids derived from the *Cannabis sativa* L. plant [19]. A body of preclinical data indicate that THC and CBD have anti-inflammatory [20,21] and antioxidant [22,23] propensity, and TLRs are cannabinoid targets [8,24]. Indeed, recent findings from our laboratory indicate that both THC and CBD target inflammatory signalling governed by TLRs in human macrophages, specifically TLR3 and TLR4 [25]. Such data, particularly with regard to THC, are important in the context of evidence linking the endocannabinoid system (ECS) with the pathogenesis of MS. Indeed, the expression of the endocannabinoid anandamide (AEA) is enhanced in CSF, lymphocytes [26], and plasma [27] of pwMS, while knock-out of the cannabinoid receptors CB_1_ [28,29,30] and CB_2_ [31], and administration of the AEA metabolising enzyme fatty acid amide hydrolase (FAAH) [30], alters the clinical progression of EAE in mice.

Cannabinoids have been an area of scientific interest in MS, particularly since the approval of nabiximols (Sativex^®^) (a complex botanical mixture, which contains CBD and THC as the most abundant cannabinoid components in addition to non-cannabinoid constituents; GW Pharmaceuticals, Cambridge, UK) for the treatment of spasticity symptoms in pwMS across the UK, EU and select other regions. To date, no studies have assessed the impact of phytocannabinoids on viral- and bacterial-like inflammatory signalling via TLR3 and TLR4 in immune cells from pwMS. In this study we sought to determine if botanically-derived purified CBD and THC (final concentration of 10 μM, delivered alone and in 1:1 combination as 10 μM:10 μM) can modulate TLR3 and TLR4 signalling in PBMCs from healthy subjects and pwMS. The data indicate that THC and CBD can act as TLR3 and TLR4 modulating drugs in primary immune cells in vitro, and offers insight on the cellular target(s) of phytocannabinoids in targeting inflammation in the context of MS.

## 2. Results

### 2.1. Demographic Data of Study Participants

The demographics of the study participants are reported in Table 1. A total of 47 subjects were included in the study, consisting of healthy control (HC) individuals (*n* = 26) and pwMS (*n* = 21). All pwMS included in the study had a relapsing–remitting (RR) form of MS. Median disease symptom duration in the MS cohort was 4.4 (2.4–5.8) years and subjects had median EDSS scores of 1.8. The median age of the 47 participants included in the study was 34.0 years (range 21–71 years in control cohort; range 22–57 years in MS cohort) and 81% of the study participants were female. Clinically significant factors pertaining to both groups are outlined in Table 1. No distinct measure to document spasticity symptoms was collated at the time of blood sample collection. Of the pwMS included in this study, five individuals were actively treated with peginterferon beta-1a, three with natalizumab, two with fingolimod, two with rituximab, and one with dimethlyfumarate, glatiramer acetate and IFN beta-1a (Table 1). Full medication use at the time of enrolment in the study is indicated in Table 1. Despite a median EDSS score of 1.8, physical and mental health summary scores derived from the MSQOL-54 questionnaire were significantly lower in pwMS when compared to the control cohort (*p* < 0.001; Table 1). Furthermore, the median QIDS-SR_16_ total score in pwMS in the study was 7.0 (Table 1), indicating mild depression in this cohort. Overall, these findings indicate that physical and mental health were significantly lower in pwMS, compared to HCs, at the time of venepuncture.

### 2.2. Time-Dependent Effect of TLR3 and TLR4 Stimulation on CXCL10, IFN-β, and TNF-α Protein Expression in PBMCs

**Figure 1:**
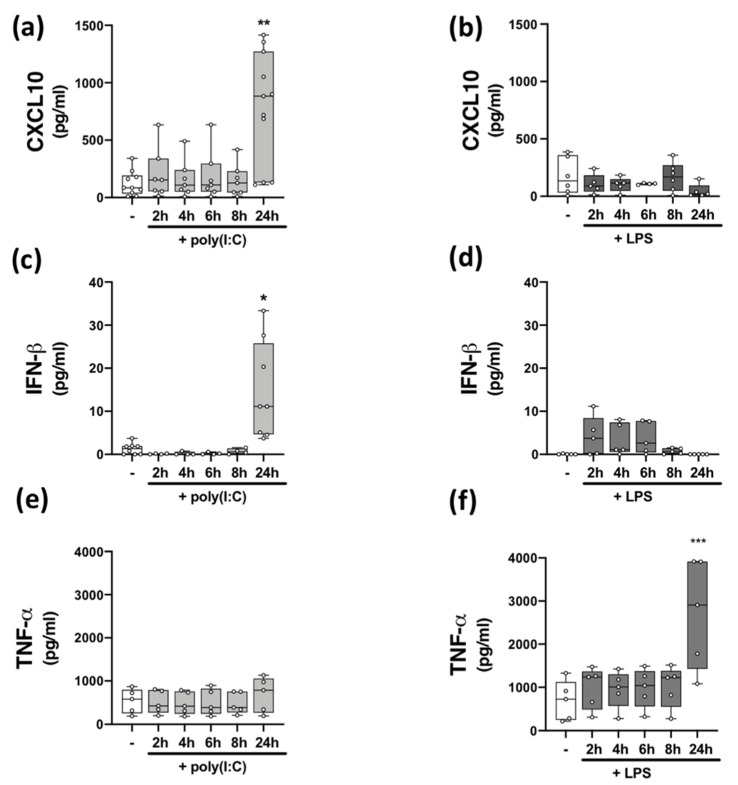
Time-dependent effect of TLR3/4 stimulation on CXCL10, IFN-β, and TNF-α expression in PBMCs from healthy volunteers. Time-dependent (2–24 h) effect of poly(I:C) (10 μg/mL) treatment on the protein expression of (**a**) CXCL10 (Kruskal–Wallis statistic = 14.4), (**c**) IFN-β (Kruskal–Wallis statistic = 20.6), and (**e**) TNF-α (*F*(5,24) = 0.33; ANOVA) in PBMCs. Time-dependent (2–24 h) effect of LPS (100 ng/mL) treatment on the protein expression of (**b**) CXCL10 (Kruskal–Wallis statistic = 3.98), (**d**) IFN-β (Kruskal–Wallis statistic = 13.4), and (**f**) TNF-α (*F*(5,24) = 6.16; ANOVA) in PBMCs. Data were tested for normality using the Shapiro–Wilk test and statistical significance determined by Kruskal–Wallis tests, with Dunn’s multiple comparison post-hoc test (for (**a**–**d**)), or one-way ANOVA with Dunnett’s multiple comparison test (for (**e**,**f**)). Data are presented as median cytokine/chemokine expression, minimum and maximum values and IQR. Symbols indicate individual data points from HCs (*n* = 5–11). * *p* < 0.05, ** *p* < 0.01 and *** *p* < 0.001 vs. cells treated with media alone.

Data from human and animal studies demonstrate that TLRs are players in MS pathogenesis [7,8,15], and neuroinflammation is linked with uncontrolled/atypical TLR signalling [32]. Innate immune responses to viral signalling via TLR3 [33] and bacterial signalling via TLR4 [34] mediates cellular inflammation. We set out to assess the impact of TLR3 and TLR4 stimulation on inflammatory responses of immune cells isolated from HCs and pwMS. We initially confirmed that PBMCs endogenously express *TLR3* and *TLR4* mRNA, with no difference in the relative expression of *TLR3* and *TLR4* mRNA determined between HCs and pwMS (Table 2). To characterize the impact of poly(I:C) and LPS stimulation on TLR3 and TLR4 signalling, respectively, we temporally assessed the impact of poly(I:C) and LPS on the production of the CXCL10 chemokine, type I IFN-β, and the pro-inflammatory cytokine TNF-α, in PBMCs from HCs (Figure 1). PBMCs were stimulated with poly(I:C) and LPS for timepoints ranging from 2–24 h and supernatants assessed for cytokine and chemokine expression. Stimulation of PBMCs with poly(I:C) promoted an increase in the expression CXCL10 (*p* < 0.01; Figure 1a) and IFN-β (*p* < 0.05; Figure 1c) at 24 h, while LPS had no significant effect on the expression of CXCL10 (Figure 1b) and IFN-β (Figure 1d). However, treatment with LPS promoted an increase in the expression of TNF-α (*p* < 0.001; Figure 1f) at 24 h. Poly(I:C) had no effect on the expression of TNF-α (Figure 1e) at each timepoint tested. This indicates that poly(I:C) promotes TLR3-induced CXCL10 and IFN-β protein expression in PBMCs, while LPS promotes TLR4-induced TNF-α expression in PBMCs, both at the 24 h timepoint.

Previous data from our laboratory suggests that TLR3 signalling is desensitized, while TLR4 signalling is exacerbated, in immune cells isolated from pwMS, when compared to PBMCs from HCs [17]. To determine the impact of disease on TLR3/4 signalling in PBMCs isolated from the current cohort of study participants, PBMCs from HCs and pwMS were treated with poly(I:C) or LPS for 24 h, supernatants harvested and assessed for protein expression of CXCL10 or IFN-β (to assess TLR3 signalling) (Table 3), and TNF-α (to assess TLR4 signalling) (Table 4). Data herein indicates that poly(I:C) significantly enhanced CXCL10 protein expression in PBMCs from control cases, when compared to HC PBMCs treated with media alone (*p* < 0.001). Two-way ANOVA analysis also indicated that poly(I:C) did not significantly increase CXCL10 protein expression in PBMCs from pwMS, when compared to PBMCs from pwMS that were treated with media alone (Table 3). Importantly, two-way ANOVA revealed a significant influence of disease status in terms of CXCL10 expression (*p* < 0.05). Indeed, post-hoc analysis determined a significant difference between poly(I:C)-treated PBMCs from HCs and poly(I:C)-treated PBMCs from pwMS, in terms of CXCL10 expression (*p* < 0.05). In terms of IFN-β protein expression, two-way ANOVA analysis indicated that poly(I:C) treatment significantly enhanced IFN-β protein expression in PBMCs from both HCs (*p* < 0.05) and pwMS (*p* < 0.001), when compared to cells treated with media alone in both cohorts (Table 3). There was no significant influence of disease on IFN-β protein expression. In terms of TLR4 signalling, LPS exposure significantly enhanced TNF-α protein expression in PBMCs from both HCs (*p* < 0.001) and pwMS (*p* < 0.001), when compared to cells treated with media alone in both cohorts (Table 4). Again, two-way ANOVA analysis revealed no significant influence of disease on TNF-α protein expression.

### 2.3. THC:CBD Inhibit TLR3-Induced CXCL10 Protein Expression in PBMCs from HCs and pwMS

We next assessed the proclivity of THC and CBD, when delivered alone and in a 1:1 combination, to modulate TLR3-induced CXCL10 release in PBMCs isolated from HCs and pwMS. It is noteworthy that the expression profile of components of the ECS, including cannabinoid receptor genes *CNR1* and *CNR2,* and ECS metabolizing enzymes *FAAH* and *MGLL*, were determined via PCR in PBMCs from both cohorts, with no significant difference in the relative expression of *CNR1*, *CNR2, FAAH,* and *MGLL* mRNA determined between both groups (Table 5).

**Figure 2:**
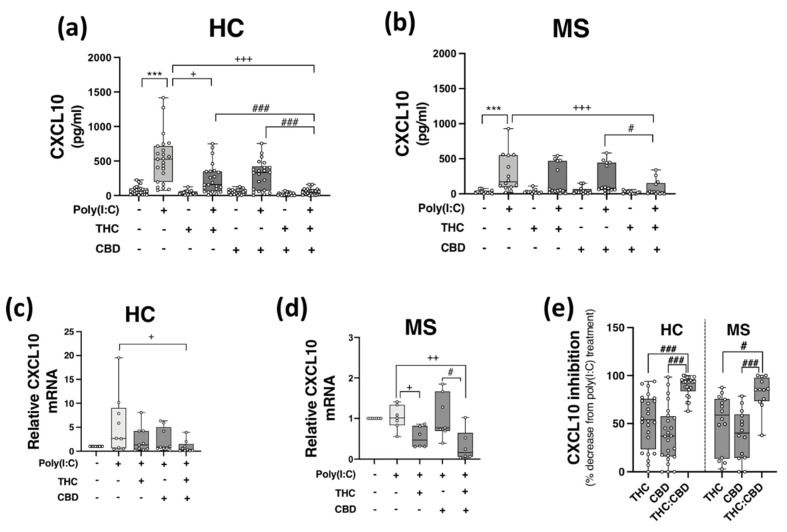
THC:CBD inhibits TLR3-induced CXCL10 in PBMCs from HC subjects and pwMS. Treatment of PBMCs from (**a**) HCs and (**b**) pwMS with poly(I:C) (10 μg/mL; 24 h) increased CXCL10 protein expression. Pre-treatment with THC (10 μM) in combination (1:1) with CBD (10 μM) (45 min pre-treatment) significantly inhibited poly(I:C)-induced CXCL10 expression in PBMCs from (**a**) HCs (Kruskal–Wallis statistic = 94.0) and (**b**) pwMS (Kruskal–Wallis statistic = 36.2). (**a**) Pre-treatment with THC (10 μM; 45 min) alone significantly inhibited poly(I:C)-induced CXCL10 expression in PBMCs from HCs (Kruskal–Wallis statistic = 94.0). Pre-treatment with a 1:1 combination of THC (10 μM) and CBD (10 μM) (45 min pre-treatment) significantly inhibited the effect of poly(I:C) on *CXCL10* mRNA expression in PBMCs from (**c**) HCs (Kruskal–Wallis statistic = 16.6), and (**d**) pwMS (Kruskal–Wallis statistic = 13.8). (**e**) Summary of the inhibitory effect of THC (10 μM), CBD (10 μM) and THC:CBD (10 μM:10 μM) on TLR3-induced CXCL10 protein expression in PBMCs from HCs and pwMS (expressed as % decrease from poly(I:C) treatment). Treatment of PBMCs from HCs with THC:CBD (10 μM:10 μM) in the presence of poly(I:C), significantly reduced CXCL10 expression when compared to PBMCs treated with poly(I:C) + THC (10 μM) and poly(I:C) + CBD (10 μM) (Kruskal–Wallis statistic = 48.5). Treatment of PBMCs from pwMS with THC:CBD (10 μM:10 μM) in the presence of poly(I:C), significantly reduced CXCL10 expression when compared to PBMCs treated with poly(I:C) + THC (10 μM) and poly(I:C) + CBD (10 μM) (Kruskal–Wallis statistic = 48.5). Statistical significance was determined by Kruskal–Wallis tests, with Dunn’s multiple comparison post-hoc test to compare means of preselected pairs of groups. Data are presented as median chemokine expression, minimum and maximum values and IQR. Symbols indicate individual data points in PBMCs from 24 HCs and 14 pwMS for protein analysis, and 10 HCs and 7 pwMS for mRNA analysis. *** *p* < 0.001 versus vehicle control group. ^+^ *p* < 0.05, ^++^ *p* < 0.01, ^+++^ *p* < 0.001 compared to cells treated with poly(I:C). ^#^ *p* < 0.05, ^###^ *p* < 0.001 vs. PBMCs treated with poly(I:C) in the presence of THC:CBD in a 1:1 combination.

PBMCs from HCs (Figure 2a,c) and pwMS (Figure 2b,d) were pre-treated (45 min) with THC (10 μM), CBD (10 μM) and THC:CBD (10 μM:10 μM) prior to poly(I:C) exposure (24 h) and assessment of CXCL10 expression. Poly(I:C) promoted a significant increase in CXCL10 release in PBMCs isolated from HCs (*p* < 0.001; Figure 2a), an effect that was significantly reduced by pre-exposure to THC and THC:CBD (*p* < 0.05, *p* < 0.001; Figure 2a). Treatment with THC:CBD significantly reduced TLR3-induced CXCL10 release when compared to treatment with either THC or CBD alone prior to poly(I:C) exposure (*p* < 0.001; Figure 2a), indicating that the combination of cannabinoids was most effective at reducing TLR3-induced CXCL10 protein expression in PBMCs isolated from HCs. Treatment with THC and CBD alone had no impact on basal CXCL10 protein expression. In terms of *CXCL10* mRNA expression, pre-exposure to THC:CBD prior to poly(I:C) exposure significantly reduced the effect of poly(I:C) treatment on *CXCL10* mRNA expression in PBMCs from HCs (*p* < 0.05; Figure 2c). Similarly, poly(I:C) significantly enhanced CXCL10 release in PBMCs from pwMS, and importantly, pre-exposure to THC:CBD significantly attenuated TLR3-induced CXCL10 protein expression in PBMCs from pwMS (*p* < 0.001; Figure 2b). In terms of *CXCL10* mRNA expression in PBMCs from pwMS, pre-exposure to THC alone or THC:CBD significantly attenuated the effect of poly(I:C) treatment on *CXCL10* mRNA expression in PBMCs from pwMS (*p* < 0.05, *p* < 0.01; Figure 2d).

Overall, data presented herein suggest that THC and CBD, when delivered in a 1:1 combination, significantly attenuate TLR3-induced inflammatory signalling associated with the expression of CXCL10 in PBMCs from both HC subjects and pwMS. Indeed, THC:CBD inhibited poly(I:C)-induced CXCL10 protein expression by 89% and 83% on average in immune cells from HCs and pwMS, respectively (Figure 2e). This inhibitory effect of the combination of phytocannabinoids on TLR3-induced CXCL10 was significantly more effective than administration of either THC or CBD alone in PBMCs from both HCs and pwMS, indicating an additive effect of their combination (Figure 2e).

### 2.4. THC:CBD Inhibit TLR3-Induced IFN-β Protein Expression in PBMCs from HCs and pwMS

**Figure 3:**
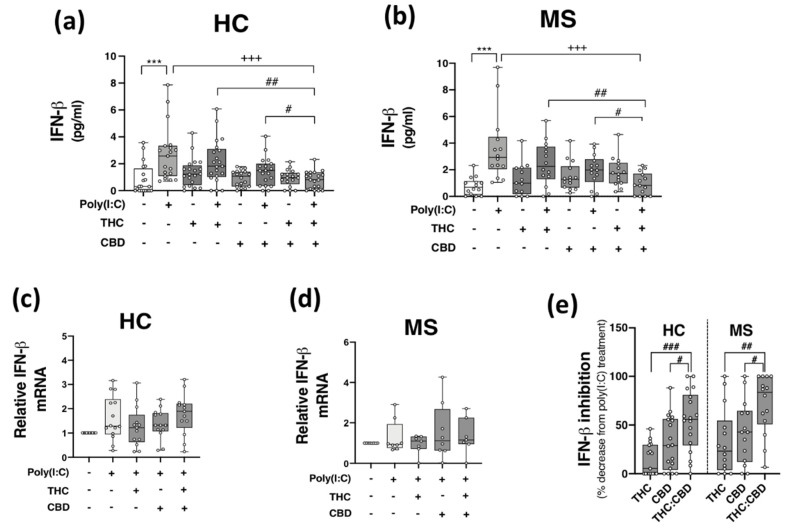
THC:CBD inhibits TLR3-induced IFN-β protein expression in PBMCs from HC subjects and pwMS. Treatment of PBMCs from (**a**) HCs and (**b**) pwMS with poly(I:C) (10 μg/mL; 24 h) increased IFN-β protein expression. Pre-treatment with THC (10 μM) and CBD (10 μM) in combination (1:1) (45 min pre-treatment) significantly inhibited poly(I:C)-induced IFN-β protein expression in PBMCs from (**a**) HCs (Kruskal–Wallis statistic = 30.4) and (**b**) pwMS (Kruskal–Wallis statistic = 30.3). Effect of poly(I:C), THC (10 μM) and CBD (10 μM) treatment on *IFN-β* mRNA expression in PBMCs from (**c**) HCs (Kruskal–Wallis statistic = 30.4), and (**d**) pwMS (Kruskal–Wallis statistic = 1.1). (**e**) Summary of the inhibitory effect of THC (10 μM), CBD (10 μM) and THC:CBD (10 μM:10 μM) on TLR3-induced IFN-β protein expression in PBMCs from HCs and pwMS (expressed as % decrease from poly(I:C) treatment). Treatment of PBMCs from HCs with THC:CBD (10 μM:10 μM) in the presence of poly(I:C), significantly inhibited IFN-β protein expression when compared to PBMCs treated with poly(I:C) + THC (10 μM) and poly(I:C) + CBD (10 μM) (Kruskal–Wallis statistic = 27.1). Treatment of PBMCs from pwMS with THC:CBD (10 μM:10 μM) in the presence of poly(I:C), significantly inhibited IFN-β protein expression when compared to PBMCs treated with poly(I:C) + THC (10 μM) and poly(I:C) + CBD (10 μM) (Kruskal–Wallis statistic = 27.1). Statistical significance was determined by Kruskal–Wallis tests, with Dunn’s multiple comparison post-hoc test to compare means of preselected pairs of groups. Data are presented as median IFN-β expression/inhibition, minimum and maximum values and IQR. Symbols indicate individual data points in PBMCs from 21 HCs and 14 pwMS for protein analysis, and 15 HCs and 8 pwMS for mRNA analysis. *** *p* < 0.001 versus vehicle control group. ^+++^ *p* < 0.001 compared to cells treated with poly(I:C). ^#^ *p* < 0.05, ^##^ *p* < 0.01 and ^###^ *p* < 0.001 vs. PBMCs treated with poly(I:C) in the presence of THC:CBD in a 1:1 combination.

Given that THC:CBD, when administered in a 1:1 combination, inhibits TLR3-induced production of the inflammatory chemokine CXCL10 in immune cells, we next assessed the proclivity of phytocannabinoids to modulate TLR3-induced type I IFN-β expression. Data presented in Figure 3 indicate that poly(I:C) treatment significantly enhanced IFN-β protein expression in PBMCs from both HCs (*p* < 0.001; Figure 3a) and pwMS (*p* < 0.001; Figure 3b). Pre-exposure to THC:CBD in a 1:1 combination, prior to poly(I:C) exposure, significantly attenuated the TLR3-induced elevation in IFN-β protein expression in PBMCs from HCs (*p* < 0.001; Figure 3a) and pwMS (*p* < 0.001; Figure 3b). These findings did not translate at the level of *IFN-β* mRNA expression, with poly(I:C), THC and CBD having no significant effect on *IFN-β* mRNA expression in PBMCs from HCs (Figure 3c) and pwMS (Figure 3d). These findings suggest that delivery of THC:CBD in a 1:1 combination, inhibits TLR3-induced IFN-β protein expression in PBMCs from both cohorts. Indeed, THC:CBD inhibited poly(I:C)-induced IFN-β protein expression by 54% and 71% on average in immune cells from HCs and pwMS, respectively (Figure 3e). The inhibitory effect of the combination of phytocannabinoids on TLR3-induced IFN-β protein expression was more effective than administration of THC or CBD alone in PBMCs from both HCs and pwMS, again, indicating an additive effect of their combination (Figure 3e).

### 2.5. Phytocannabinoids Exacerbate TLR4-Induced TNF-α Expression in PBMCs from HCs and pwMS

**Figure 4:**
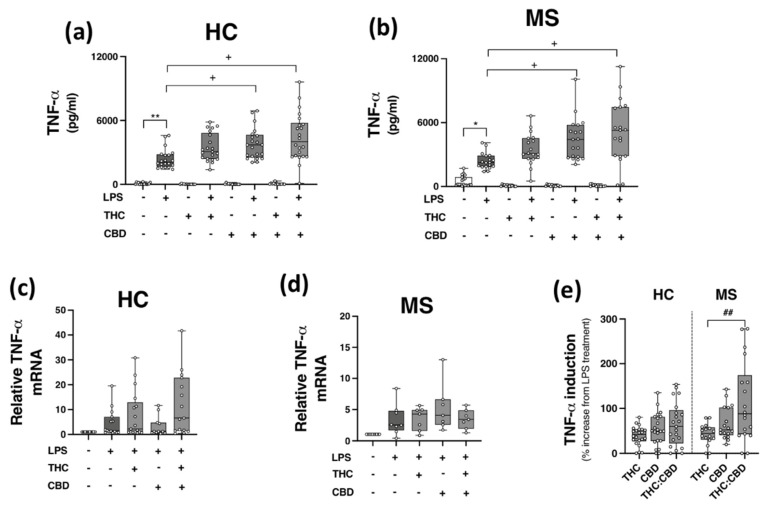
CBD and THC:CBD exacerbate TLR4-induced TNF-α expression in PBMCs from HC subjects and pwMS. Treatment of PBMCs from (**a**) HCs, and (**b**) pwMS with LPS (100 ng/mL; 24 h) increased TNF-α protein expression. Pre-treatment with CBD (10 μM) alone, and in combination (1:1) with THC (10 μM) (45 min pre-treatment), significantly exacerbated LPS-induced TNF-α expression in PBMCs from (**a**) HCs (Kruskal–Wallis statistic = 106.9), and (**b**) pwMS (Kruskal–Wallis statistic = 101.7). Effect of LPS, THC (10 μM) and CBD (10 μM) treatment on *TNF-α* mRNA expression in PBMCs from (**c**) HCs (Kruskal–Wallis statistic = 14.6), and (**d**) pwMS (Kruskal–Wallis statistic = 17.9). (**e**) Summary of the stimulatory effect of THC (10 μM), CBD (10 μM), and THC:CBD (10 μM:10 μM) on TLR4-induced TNF-α protein expression in PBMCs from HCs and pwMS (expressed as % increase from LPS treatment). Treatment of PBMCs from pwMS with THC:CBD (10 μM:10 μM) in the presence of LPS, significantly increased TNF-α protein expression when compared to PBMCs treated with LPS + THC (10 μM) (Kruskal–Wallis statistic = 14.0). Statistical significance was determined by Kruskal–Wallis tests, with Dunn’s multiple comparison post-hoc test to compare means of preselected pairs of groups. Data are presented as median cytokine expression, minimum and maximum values and IQR. Symbols indicate individual data points in PBMCs from 22 HCs and 19 pwMS for protein analysis, and 18 HCs and 10 pwMS for mRNA analysis. * *p* < 0.05, ** *p* < 0.01 vs. vehicle control group. ^+^ *p* < 0.05 compared to cells treated with LPS. ^##^ *p* < 0.01 vs. PBMCs treated with LPS in the presence of THC:CBD in a 1:1 combination.

Given the inhibitory effects of THC:CBD on TLR3 signalling to CXCL10 and IFN-β in PBMCs, in addition to evidence linking TLR4 to MS pathogenesis [15,16], we next set out to determine the proclivity of THC and CBD, when delivered alone and in a 1:1 combination, to modulate TLR4-induced TNF-α protein expression in PBMCs isolated from HCs and pwMS. PBMCs from HCs (Figure 4a,c) and pwMS (Figure 4b,d) were pre-treated (45 min) with THC (10 μM), CBD (10 μM) and THC:CBD (10 μM:10 μM) prior to LPS exposure (24 h), and TNF-α protein expression assessed. Data presented in Figure 4a indicates that LPS enhanced TNF-α release in PBMCs from control cases (*p* < 0.01); surprisingly, CBD (*p* < 0.05) and THC:CBD (*p* < 0.05) significantly exacerbated LPS-induced TNF-α protein expression. A similar pattern was determined in PBMCs from pwMS, with CBD and THC:CBD significantly exacerbating LPS-induced TNF-α protein expression (*p* < 0.05; Figure 4b). In terms of *TNF-α* mRNA expression, no statistical significance was determined in terms of the impact of phytocannabinoids on *TNF-α* mRNA expression in PBMCs from HCs (Figure 4c) and pwMS (Figure 4d). This suggests that PBMCs from HCs and pwMS respond to LPS in a similar pattern, in terms of TNF-α protein expression. Data presented in Figure 4 indicates that CBD and THC:CBD potentiate TLR4-induced signalling mechanisms to promote TNF-α protein expression in PBMCs from both HC subjects and pwMS. Indeed, data presented in Figure 4e indicate that THC:CBD potentiated LPS-induced TNF-α protein expression by 65% and 115% on average in PBMCs from both HCs and pwMS, respectively.

### 2.6. THC and CBD, When Delivered Alone and in 1:1 Combination, Are Not Cytotoxic to PBMCs from HCs and pwMS

**Figure 5:**
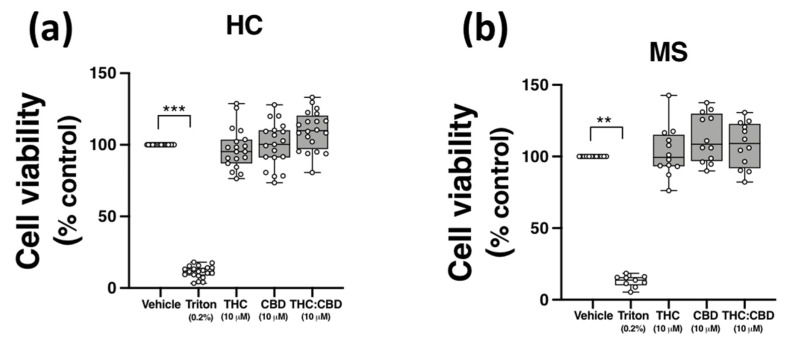
THC and CBD are not cytotoxic in PBMCs from HCs and pwMS. MTT assay of PBMCs from (**a**) HCs, and (**b**) pwMS treated (for 24 h) with THC (10 μM) alone, CBD (10 μM) alone and THC:CBD in a 1:1 combination (10 μM:10 μM). The number of control cells, i.e., viable cells exposed to vehicle (0.1% ethanol), was defined as 100% in each participant. Triton X-100 (0.2% for 10 min) treatment was used as a positive control. Kruskal–Wallis statistic = 54.5 in HC samples. Kruskal–Wallis statistic = 25.1 in MS samples. Data are presented as median viability values expressed as % control, minimum and maximum values and IQR. Symbols indicate individual data points in PBMCs from 20 HCs and 12 pwMS. Statistical significance was determined by Kruskal–Wallis tests, with Dunn’s multiple comparison post-hoc test. ** *p* < 0.01, *** *p* < 0.001 vs. vehicle control group.

Evidence from in vitro studies indicate that THC and CBD have anti-inflammatory efficacy at a concentration of 10 μM [21,35,36,37]; however, THC and CBD can also exert cytotoxic effects in vitro [38,39]. To confirm that the effect of THC and CBD on TLR3-induced CXCL10/IFN-β, and TLR4-induced TNF-α expression, were not due to cytotoxicity, the effect of THC and CBD, delivered alone and in 1:1 combination, on the viability of PBMCs was determined using MTT assays. PBMCs were incubated with ethanol vehicle control (0.1%), THC (10 μM), CBD (10 μM), or a combination of both phytocannabinoids (1:1 ratio; 10 μM final concentration for each compound) for 24 h, and the impact of phytocannabinoid treatment on the proliferation of PBMCs was quantified using MTT. Data presented in Figure 5 demonstrates that no cytotoxicity was determined following treatment of PBMCs from HCs (Figure 5a) and pwMS (Figure 5b) with CBD and THC (when delivered alone and as a 1:1 ratio) at the final concentration of 10 μM. Triton X-100 (0.2%) treatment reduced cell viability (*p* < 0.01, *p* < 0.001; Figure 5a,b).

### 2.7. Plasma C-Reactive Protein (CRP) and Haematological Parameters in HC and MS Cohorts

Given the association between neurological symptoms and infection [40], we also assessed the levels of the inflammatory reactant CRP in plasma isolated from the HC and MS groups. No significant difference (*p* = 0.57) in plasma CRP protein concentration was determined between groups (see Supplementary Materials Table S1). Detection rates and median values of plasma CRP are indicated in Supplementary Table S1. In addition, no significant difference in plasma CXCL10, IFN-β and TNF-α concentrations were determined between both cohorts (data not shown). Following venepuncture, venous blood was also assessed for total white blood (WBC) count, red blood cell (RBC) count, platelet count (PLT), haemoglobin (HGB), haematocrit (HCT; %), mean corpuscular volume (MCV), mean corpuscular HGB (MCH), and mean corpuscular HGB concentration (MCHC) using Sysmex analysis. Data presented in Supplementary Table S2 indicates no significant difference in haematological parameters in samples from HCs and pwMS.

## 3. Discussion

This study set out to determine if the phytocannabinoids THC and CBD, when delivered alone and in a 1:1 combination, target TLR3 and TLR4 signalling in primary immune cells isolated from HCs and/or pwMS. The novel finding is that when THC and CBD are administered in combination (1:1), at a concentration of 10 μM:10 μM, the phytocannabinoids are effective inhibitors of TLR3 induction of the chemokine CXCL10 and type I IFN-β, in PBMCs from both MS and control cohorts. In contrast, when PBMCs from both HCs and pwMS are treated with the combination of phytocannabinoids (THC:CBD in a 1:1 combination at 10 μM:10 μM), in addition to treatment with CBD alone at 10 μM, the cannabinoids exacerbated TLR4-induced TNF-α production in PBMCs. Treatment of PBMCs with THC and CBD at 10 μM (delivered alone and in 1:1 combination) did not alter cell viability in either HC or MS groups. The study also determined the characteristics that exist between pwMS and HCs in parameters at the time of blood sample collection, including QOL (physical and mental health scores), depressive symptomatology, relative expression of TLR3 and TLR4 and the expression of components of the ECS in PBMCs. Data herein indicate that both physical and mental health scores were lower in pwMS, compared to HCs, and no alterations in the relative expression of *TLR3*, *TLR4, CNR1*, *CNR2*, *FAAH,* and *MGLL* mRNA was determined in PBMCs from HCs and pwMS.

Previous findings from our laboratory indicate that THC and CBD exert anti-inflammatory propensity by targeting the TLR3-IFN-β/CXCL10 and TLR4-TNF-α signalling axes in human THP-1-differentiated macrophages [25]. Both adaptive and innate arms of the immune system contribute to MS pathogenesis [41,42], and given the role of lymphocytes [43], monocytes [44] and dendritic cells (DCs) [45] in MS pathogenesis, we aimed to translate our findings in THP-1 macrophages to a clinical context by assessing the effect of phytocannabinoids on TLR signalling in PBMCs isolated from whole blood in pwMS and controls. Several immunomodulatory disease modifying therapies (DMTs) have been approved in MS, which target the peripheral immune system. Human PBMCs consist of a population of lymphocytes (T/B cells and NK cells), DCs and monocytes [46], and express a repertoire of TLRs (TLR1-10) [47] to mediate cellular responses to infection. We particularly targeted TLR3 and TLR4 signalling given their role in neuroinflammation in MS [7,15,48], with knockout of TLRs and their adaptors either exacerbating [9,10] or ameliorating [9,12,13] the progression of EAE. In terms of TLR3 and TLR4, the expression of both receptors is elevated in active MS brain lesions [15] and the expression of TLR4 is upregulated in mononuclear cells from pwMS [16]. Our findings demonstrate that both TLR3 and TLR4 are expressed in PBMCs and respond to poly(I:C) and LPS to promote CXCL10/IFN-β and TNF-α expression, respectively. This is consistent with findings elsewhere [49,50,51]. Data presented herein suggest that MS does not impact the relative expression profile of TLR3 and TLR4 in PBMCs, and also suggests that PBMCs respond to LPS and poly(I:C) in a similar manner. Furthermore, we have previously shown that PBMCs from pwMS are hypersensitive to LPS treatment in terms TNF-α expression [17]. This finding was not replicated in the MS cohort investigated in the current study, and may reflect the impact of DMT use, and stage of disease progression, on cellular responses to TLR4 activation.

TLRs are known cannabinoid targets, and TLR signalling can be modulated by phytocannabinoids, synthetic cannabinoids and endocannabinoids [8]. We report that THC and CBD in a 1:1 combination ameliorates TLR3-induced signalling controlling the production of IFN-β and CXCL10, while THC:CBD exacerbates TLR4-induced TNF-α expression. Elsewhere, in terms of the impact of phytocannabinoids on TLR4 signalling, THC (10 μM) and CBD (5 μM, 10 μM) have been shown to inhibit TLR4-induced IL-1β production in microglia [35]. CBD (3 μM) also reduces TLR4-induced oxidative stress in mesenchymal stromal cells [52], while tetrahydrocannabivarin (THCV; 1 μM) inhibits TLR4 inflammatory signalling in macrophages [53]. In terms of TLR3 signalling, we have previously shown that THC and CBD (both at 10 μM) inhibit TLR3-induced inflammatory signalling in macrophages [25], which is consistent with the effects of several phytocannabinoids including CBD, THCV, cannabigerol (CBG), cannabichromene (CBC), and cannabigevarin (CBGV) (all in the 5–20 μM range) on TLR3 signalling in keratinocytes [21]. In support of the propensity of CBD to target TLR3 signalling, in vivo administration of CBD (1 mg/kg; intraperitoneal administration) to mice during the peripubertal period ameliorates hyperlocomotion following prenatal exposure to poly(I:C) [54], while in vivo administration of CBD (10 mg/kg intraperitoneal administration for three weeks, twice a day, at 12 h intervals) to rats attenuates cognitive/social interaction deficits induced by pre-natal poly(I:C) exposure [55]. Data presented in the current study provides further evidence that phytocannabinoids target bacterial and viral signalling via TLR4 and TLR3 to impact inflammatory signalling.

Data presented herein identifies that THC and CBD in a 1:1 combination ameliorates TLR3-induced signalling controlling the production of IFN-β and CXCL10. TLR3 signals via the TRIF adaptor to promote downstream signalling via IRF3 to regulate the transcription of IFNs and other inflammatory mediators such as CXCL10 [56,57]. CXCL10 is an IFN inducible protein, and both IFN-β and CXCL10 exert a range of immunomodulatory effects [58,59]. Indeed, CXCL10 binds to its receptor CXCR3 to mediate the recruitment of lymphocytes, monocytes, and NK cells to inflamed tissues [60,61]. The inhibitory effect of THC:CBD on CXCL10 expression in PBMCs from both HCs and pwMS is consistent with the effect of these phytocannabinoids in macrophages [25], and is significant given that CXCL10 is elevated in CSF [62], PBMCs [63], and serum [64] isolated from pwMS, with elevations in this chemokine associated with increased numbers of leukocytes in CSF [62]. Monocytes and DCs are producers of CXCL10 [61], and our findings suggests that THC and CBD can act on monocytes and DCs in the PBMC population to exert anti-inflammatory propensity.

IFN-β is a first-line DMT for RRMS [65], with an array of anti-viral and immunosuppressive actions that include the downregulation of cytokine expression [66] and the inhibition of immune cell passage across the blood brain barrier [67]. Our results indicate that THC:CBD negatively regulate TLR3-induced IFN-β expression in PBMCs, which is again consistent with previous data from our group demonstrating that THC (10 μM) and CBD (10 μM) inhibit TLR3- and TLR4-induced IFN-β expression in human macrophages [25]. This is also consistent with the inhibitory effect of THC and CBD (both at 10 μM) on TLR4-induced IFN-β expression in murine microglia [35]. Given that the transcription of IFN-β is controlled by IRF3 [68], and that nuclear sequestration of IRF3 is inhibited by THC:CBD (1:1, both at 10 μM) [25], it is likely that phytocannabinoids target the upstream phosphorylation and nuclear sequestration of IRF3 in response to treatment with TLR3 in PBMCs from HCs and pwMS. This will be the focus of further studies. IFN-β acts at cell surface receptors (designated IFNAR) to promote signalling via the Janus kinase-signal transducers and activators of transcription (JAK-STAT) to regulate IFN-inducible genes [69]. Indeed, IFN-β has the proclivity to upregulate CXCR3 chemokines such as CXCL10 in PBMCs [70]. Hence, it is reasonable to suggest that the combination of THC and CBD can inhibit IFN-β-dependent pro-inflammatory processes by indirectly negating IFN-β-induced CXCL10 expression in PBMCs. Kozela and colleagues (2010) identified STAT1 phosphorylation as a target for both THC and CBD following LPS treatment [35], suggesting that JAK-STAT signalling may mediate the mechanism by which THC and CBD regulate IFN-β-dependent inflammatory processes in PBMCs from HCs and pwMS.

Transcriptional production of TNF-α following TLR4 activation is controlled by several transcription factors including members of the NF-κB and activator protein-1 (AP-1) protein families [71]. LPS can signal via the MyD88 adaptor to regulate NF-κB and MAPKs to control inflammatory cytokine/chemokine expression, or, alternatively, via MyD88-independent pathways (via TRIF) to regulate NF-κB and IRFs, and, hence, the production of IFNs and cytokines including TNF-α [72]. It was surprising to note that THC:CBD, and CBD alone, exacerbated LPS-induced TNF-α expression in PBMCs from both HCs and pwMS. Further experiments are required to determine the functional impact of the effect of THC and CBD on LPS-induced TNF-α expression, and the mechanistic basis to this remains to be elucidated both in vitro and in vivo. However, it has been shown that cannabinoids can enhance the activation of NF-κB (including THC at 5 μM in vitro) [73], MAPKs (including THC at 5 μM in vitro) [73,74], and AP-1 (including THC at 10–15 mg/kg in vivo) [75], and, indeed, promote TNF-α expression (including the selective CB_1_ and CB_2_ agonists, ACEA and JWH-056 at 0.5 μM in vitro) [76]. Hence, it is plausible that THC and CBD, particularly when delivered in a 1:1 combination, may potentiate LPS-induced MAPK and(or) NF-κB signalling in PBMCs. Furthermore, given evidence that the expression of TLR4 is upregulated in immune cells from pwMS [16], it is plausible that the effect of THC:CBD on LPS-induced TNF-α may reflect disease-related changes in the relative expression and(or) function of TLR4 in PBMCs. However, it should be noted that TLR4 was detected in PBMCs in the current study, which was expected given its expression on monocytes, macrophages and mature DCs [77], and no difference in the relative expression of this receptor was determined in PBMCs isolated from the control and MS groups.

This study compared the anti-inflammatory potential of THC and CBD when delivered alone versus delivery in a 1:1 combination, and highlights that the combination of phytocannabinoids was more effective in modulating TLR3 and TLR4 signalling in PBMCs. In terms of CXCL10, PBMCs from HCs and pwMS responded to THC:CBD combination treatment by significantly reducing TLR3-induced CXCL10 protein expression by 89% and 83%, respectively. Indeed, THC alone reduced TLR3-induced CXCL10 expression by 53% and 51% in PBMCs from HCs and pwMS, respectively, while CBD ameliorated TLR3-induced CXCL10 by 39% and 38% in PBMCs from HCs and pwMS, respectively, indicating that the anti-inflammatory propensity of the phytocannabinoids investigated in this study was more evident following THC and CBD treatment in combination, compared to their delivery alone. A similar pattern was seen in terms of THC:CBD inhibition of TLR3-induced IFN-β (54% and 71% in HCs and pwMS), compared to THC alone (14% and 33% in HCs and pwMS) and CBD alone (32% and 43% in HCs and pwMS). In support of this, the combination of botanical extracts of CBD and THC (1:1 combination of CBD and THC, in addition to CBG, CBC, and other phytocannabinoids) has been shown to be more effective in the restoration of motor function in the Theiler’s murine encephalomyelitis virus-induced demyelination model, when compared to treatment with THC and CBD alone [78]. This argues in favour of the therapeutic potential of THC and CBD when delivered in a 1:1 combination. It is important to note that THC:CBD in combination (65% and 115% in HCs and pwMS) exacerbated TLR4-induced TNF-α protein expression in PBMCs to a greater extent than THC (39% and 42% in HCs and pwMS) and CBD (54% and 66% in HCs and pwMS) alone. A comparison of the effect of THC and CBD alone, versus THC:CBD 1:1, on LPS-induced MAPK and NF-κB signalling in PBMCs warrants full investigation. Furthermore, TNF-α production is associated with cell death mechanisms [79], and given that the phytocannabinoids investigated in this study have also been associated with toxicity at similar concentrations to that used in the present study [80,81], we assessed the impact of THC and CBD (alone and 1:1 combination at 10 μM) on primary PBMC viability and determined that THC, CBD and THC:CBD did not negatively impact the viability of PBMCs from both HCs and pwMS.

Our findings, particularly regarding THC, are important in the context of the ECS in MS, given reports demonstrating changes in the expression of endocannabinoids in CSF, lymphocytes [26] and plasma [27] of pwMS, while the expression of FAAH and CB_2_ is altered in whole blood of pwMS [27]. Furthermore, CB_1_ [28,30] and CB_2_ [31] knock-out exacerbates EAE progression, while mice lacking FAAH have reduced EAE clinical scores [30]. Given these findings, we assessed the relative expression of *CNR1*, *CNR2*, *FAAH,* and *MGLL* mRNA in PBMCs, and observed no effect of disease on the expression of these components of the ECS. Conclusions relating to MS pathophysiology from such findings should be made with caution, given that our assessment of the ECS in PBMCs was limited to a single method of analysis. However, it is unlikely that rearrangement of the ECS involving the cannabinoid receptors and endocannabinoid metabolising enzymes has taken place in PBMCs isolated from the MS cohort investigated herein. The role of the ECS in contributing to the effect(s) of THC and CBD in PBMCs from patients is an important consideration. However, CBD demonstrates minimal agonist activity (and low affinity) for both CB_1_ and CB_2_ [82,83], while THC is a CB_1/2_ partial agonist [84,85]. CB_1_- and CB_2_-independent effects of THC [35,86] and CBD [35,87] have been published, and data from our laboratory supports this in terms of THC/CBD modulation of TLR3 and TLR4 signalling in human macrophages [25]. It is important to note that at the time of recruitment, two pwMS were taking Gilenya^®^ (Fingolimod; FTY720), an approved DMT for RRMS [88]. FTY720 acts by targeting the spingosine-1-phosphate receptor (S1PR) to attenuate egress of lymphocytes from lymph nodes. S1PRs can modulate TLR4-induced signalling in glia [89], and importantly, evidence indicates that FTY720 and sphingosine can act as antagonists at CB_1_ [90], with CB_2_ agonists having the proclivity to modulate the metabolism of sphingolipids [91]. Overall, further studies are required to determine the pharmacological targets of THC and CBD in modulating TLR3/4 signalling in PBMCs.

The current study investigated QOL (physical/mental health summary scores) and depressive symptomatology at the time of blood draw. Overall, QOL is reduced in pwMS [92] and pwMS also exhibit high levels of anxiety and symptoms of depression [93,94]. Data presented herein support this demonstrating that both physical and mental health summary scores derived from the MSQOL-54 questionnaire were significantly lower in pwMS when compared to the control cohort, despite a median EDSS score of 1.8. Although both alterations in TLR signalling [95] and the ECS [96] have been associated with QOL indices such as mental health in select populations, such as patients with major depressive disorder and trauma-injury survivors, respectively, we determined no significant correlation between the expression of TLR3/4, or components of the ECS, and the QOL readings made in this study cohort of pwMS (data not shown). It is important to note that bacterial infections are associated with an exacerbation of MS symptoms [97], and bacterial infection can promote an increase in levels of CRP [98]. However, no significant differences in plasma concentrations of CRP were identified between the cohorts in this study. In support of this, we found no change in WBC counts between controls and pwMS in our haematological analysis at the time of blood draw. Hence, it is likely that participants had no underlying infection at the time of blood sample collection that may modulate the cellular responses reported herein to LPS/poly(I:C) agonist exposure. Indeed, no significant change in the relative expression of *TLR3* and *TLR4* were determined at baseline in cells from HCs and pwMS.

Caution must be taken when extrapolating the effects of phytocannabinoids in vitro to clinical use of cannabinoid-based therapies in vivo. Although the concentrations THC and CBD employed in vitro in the current study are considered supraphysiological, the 10 μM dose was not toxic to primary PBMCs, and is also consistent with in vitro assessment of phytocannabinoids in inflammatory studies elsewhere [35,99]. The precise pharmacological target(s) for THC and CBD in modulating TLR3/4 signalling in PBMCs is unclear and warrants full investigation.

In conclusion, the precise cellular mechanism(s) of action of both CBD and THC underlying therapeutic efficacy in immune cells from pwMS is unclear. This study gives mechanistic insight to the modulatory effects of THC and CBD on inflammatory signalling at a cellular level in PBMCs from both HCs and pwMS. We have identified that THC and CBD, particularly when delivered in vitro in a 1:1 combination at [10 μM], are effective inhibitors of TLR3 signalling controlling the production of CXCL10 and IFN-β in PBMCs from HCs and pwMS. In addition, the phytocannabinoids demonstrated propensity to exacerbate TLR4 signalling controlling the production of TNF-α in PBMCs from both study cohorts. No significant difference in the relative expression of *CNR1*, *CNR2, FAAH,* and *MGLL* mRNA was determined in PBMCs isolated from both groups, suggesting that the expression of components of the ECS in immune cells was not altered in pwMS assessed in the study. Overall, the study provides new insight into the cellular effects of CBD and THC, when administered alone and as a 1:1 combination, in modulating TLR signalling in primary immune cells from pwMS and controls. This is important given the role of TLRs in innate immunity and inflammation.

## 4. Materials and Methods

### 4.1. Study Participants and Blood Samples

HCs and pwMS attending clinics at Beaumont Hospital, Dublin, Ireland, were recruited to this study (Table 1). Written informed consent was obtained from each participant and the study received ethical approval from the Beaumont Hospital Ethics (Medical Research; Ref: 16/47) and the Faculty of Health Sciences Research Ethics Committee, Trinity College Dublin, Ireland (Ref: 160502). The study was conducted in accordance with the Declaration of Helsinki. The recruitment of pwMS into the study was via a Consultant Neurologist and all pwMS were clinically stable with a RR form of MS as defined by the revised McDonald criteria [100], including patient history, clinical signs and spasticity symptoms, physical examination, and adjunctive diagnostic tools including MRI. Some pwMS were on immunomodulatory treatment including peginterferon beta-1a, natalizumab, and fingolimod (presented in Table 1). HCs with no history of cardiovascular, respiratory or degenerative disease were included. Details of participant demographics are presented in Table 1. PBMCs were prepared from venous whole blood samples by way of venepuncture (max 50 mL per donor collected in EDTA tubes) from HC participants (median age 31.5 years; *n* = 26) and pwMS (median age 37.0 years; *n* = 21) by density separation over Lymphoprep™ (Axis-Shield, Oslo, Norway). Disease severity was scored at time of collection by a Neurologist using the Expanded Disability Status Scale (EDSS) scores. PBMCs were plated (1 × 10^6^ cells/mL) on 6- or 24-well plates. Plasma samples were separated following centrifugation, aliquoted and stored at −80 °C for subsequent analysis via ELISA.

### 4.2. CRP Measurement in Plasma

Plasma samples from HCs and pwMS were analysed for concentrations of CRP by ELISA, according to manufacturer’s instructions (Duoset, R&D Systems, Abingdon, UK).

### 4.3. Blood Counts

At the time of venepuncture, an automated haematology system was used to complete blood counts. Several components were determined including white blood cells (WBCs), red blood cells (RBCs), haemoglobin (HGB), haematocrit (HCT), mean corpuscular volume (MCV), mean corpuscular haemoglobin (MCH), mean corpuscular haemoglobin concentration (MCHC), and platelet (PLT) number. Analysis was conducted using 25 mL whole blood with a Sysmex KX-21N analyser (Sysmex Europe GmbH, Norderstedt, Germany).

### 4.4. Materials

Plant-derived, highly-purified CBD and THC were provided by GW Research Ltd. (Cambridge, UK) in ethanol.

### 4.5. Cytokine Analysis in PBMC Culture Supernatants

PBMCs (1 × 10^6^ cells/mL) were maintained in culture for at least 2 h in 24-well plates. PBMCs were pre-exposed (45 min) to plant-derived, highly-purified CBD (10 μM), THC (10 μM) or THC:CBD combinations (10 μM:10 μM) (GW Research Ltd., Cambridge, UK) prior to treatment with the TLR4 agonist LPS (100 ng/mL) (Enzo Life Sciences, Inc., Farmingdale, NY, USA) or the TLR3 agonist poly(I:C) (10 μg/mL) (Invivogen) for 4 h (mRNA assessment) or 2–24 h (protein assessment). Control wells were incubated with RPMI media or RPMI media containing ethanol (0.1%) as vehicle control. The concentration of both THC and CBD used have been recently shown to modulate TLR3/4 signalling in macrophages [25], and is consistent with the concentrations of these phytocannabinoids used in inflammatory studies [21,35,36,37]. Supernatants were assayed for CXCL10, IFN-β and TNF-α concentration by ELISA, according to manufacturer’s instructions (Duoset, R&D Systems, Abingdon, UK).

### 4.6. Quantitative Real-Time PCR

PBMCs (2 × 10^6^ cells/mL) were seeded in 6-well plates and were maintained in culture for at least 2 h. PBMCs were pre-exposed (45 min) to THC (10 μM), CBD (10 μM), or a combination of both (1:1 ratio; each cannabinoid at 10 μM) prior to LPS (100 ng/mL; 4 h) or poly(I:C) (10 μg/mL; 4 h) exposure. Control wells were incubated with RPMI media or RPMI media containing ethanol (0.1%). RNA was extracted from PBMCs using a NucleoSpin^®^ RNAII isolation kit (Macherey-Nagel Inc., Dueren, Germany). The concentration of RNA was determined using a UV/Vis spectrophotometer. cDNA synthesis was performed on 1 μg RNA using a High Capacity cDNA RT Kit (Applied Biosystems, Foster City, CA, USA), according to the manufacturer’s instructions. Equal amounts of cDNA were used for PCR amplification. Real-time PCR primers were delivered as “Taqman^®^ Gene Expression Assays” containing forward and reverse primers, and a FAM-labeled MGB Taqman probe for each gene (Applied Biosystems). Primers used were as follows: TLR3, TLR4, CXCL10, IFN-β, CNR1, CNR2, TNF-α, FAAH, and MGLL (Taqman^®^ Gene Expression Assay no. Hs00152933_m1, Hs00152939_m1, Hs00171042_m1, Hs00171042_m1, Hs00275634_m1, Hs00361490_m1 and Hs01113624_g1, Hs01038664_m1, and Hs00996004_m1, respectively). cDNA (1:4 dilution) was prepared and real-time PCR performed using Applied Biosystems 7300 Real-time PCR System. cDNA was mixed with qPCR™ Mastermix Plus (Applied Biosystems) and the respective gene assay in a 25 μL volume (10 μL of diluted cDNA, 12.5 μL Taqman^®^ Universal PCR Mastermix, 1.25 μL target primer and 1.25 μL 18S rRNA). Eukaryotic 18S rRNA was used as an endogenous control and expression was conducted using a gene expression assay containing forward and reverse primers, and a VIC-labelled MGB Taqman probe (#4319413E; Applied Biosystems). Samples were run in duplicate and 40 cycles were run as follows: 10 min at 95 °C and for each cycle, 15 s at 95 °C and 1 min at 60 °C. Gene expression was calculated relative to the endogenous control and analysis was performed using the 2^−ΔΔCT^ method. For the assessment of the constitutive expression of *TLR3*, *TLR4*, *CNR1*, *CNR2*, *FAAH,* and *MGLL* mRNA in PBMCs from HC cases and pwMS, the average delta cycle threshold (Ct) values are presented.

### 4.7. Cell Viability Assay

PBMCs (0.1 x 10^4^ cells/well) were seeded in 96-well plates for 1 h and incubated with sterile ethanol (0.1%), THC (10 μM), CBD (10 μM), or a combination of both phytocannabinoids (1:1 ratio; 10 μM final concentration for each compound) (GW Research Ltd., Cambridge, UK) for 24 h. Proliferation of PBMCs was quantified using MTT (3-(4,5 dimethylthiazol-2-yl)-2,5-diphenyltetrazolium bromide) assay, as previously described [25].

### 4.8. QOL and Depressive Symptomatology

The QOL of participants was assessed at the time of blood draw using the MS Quality of Life-54 (MSQOL-54) questionnaire generating a physical and mental health composite score [101]. The MSQOL-54 is a self-report questionnaire and was completed by subjects without any additional assistance. Subjects also completed the self-rated 16-item Quick Inventory of Depressive Symptomatology (QIDS-SR_16_) questionnaire at the time of blood draw. The total QIDS-SR_16_ score (0–27) was blindly generated for pwMS and HCs, producing a quantitative result corresponding to depression severity (0–5 = none; 6–10 = mild; 11–15 = moderate; 16–20 = severe; and 21–27 = very severe). Study participants attempted and self-administered all the questions. QIDS-SR_16_ has been implemented in other studies reviewing depressive co-morbidity [102].

### 4.9. Statistical Analyses

All data were analysed using GraphPad Prism (version 9.0.0). Data were tested for normality using the Shapiro–Wilk test. Outlier tests were performed using the ROUT or Tukey’s 1.5 × IQR tests for normal and non-normal data, respectively. If data were normally distributed, parametric testing was used via student’s *t*-test or one-way ANOVA followed by post-hoc Dunnett’s multiple comparisons test. If data were not normally distributed, non-parametric testing was employed using the Mann–Whitney U test or the Kruskal–Wallis test followed by Dunn’s multiple comparisons test. Two-way ANOVA followed by Bonferroni’s post-hoc test was performed when there was more than one variable to be analysed. Data are expressed as mean ± standard error of the mean (SEM) for data that are normally distributed or as box and whisker plots with minimum/maximum values and interquartile range (IQR) for data that are not normally distributed. Symbols indicate individual data points. Within each experiment, duplicate/triplicate determinations were performed for each condition/drug treatment. When analysis indicated significance (*p* < 0.05), post-hoc Dunnett’s or Dunn’s multiple comparison tests were used to compare means of preselected pairs of groups.

## Figures and Tables

**Table 1:** Demographics.

Characteristics	HC (*n* = 26)	MS (*n* = 21)	*p* Value
Age, years, median (range)	31.5 (25.0–40.8)	37.0 (30.5–45.0)	0.1543
Gender			
Female, *n*	20	18	
Male, *n*	6	3	
EDSS, median (range)	n/a	1.8 (1.0–3.0)	
Disease duration, years, median (range)	n/a	4.4 (2.4–5.8)	
MS-QOL54 composite score			
Physical health, median (range)	94.7 (89.9–95.8)	71.8 (49.0–82.8)	<0.001 ***
Mental health, median (range)	90.8 (88.0–94.9)	73.4 (41.3–85.1)	<0.001 ***
QIDS-SR_16_ score	2.0 (1.0–4.0)	7.0 (3.0–11.5)	<0.001 ***
Symptom reported during study			
Blood disorder, *n*	1	1	
Thyroid disease, *n*	2	-	
Non-MS autoimmune disease, *n*	1	1	
Allergies, *n*	4	4	
Asthma, *n*	1	1	
Infection, *n*	1	2	
Epilepsy, *n*	-	2	
Anxiety/depression, *n*	2	2	
Overactive bladder, *n*	-	2	
Kidney disease, *n*	-	1	
MS medication use in MS group			
Peginterferon beta-1a (Plegridy^®^), *n*	-	5	
Natalizumab (Tysabri^®^), *n*	-	3	
Fingolimod (Gilenya^®^), *n*	-	2	
Rituximab (Rituxan^®^), *n*	-	2	
Dimethylfumarate (Tecfidera^®^), *n*	-	1	
Interferon beta-1a (Avonex^®^), *n*	-	1	
Glatiramer acetate (Copaxone^®^), *n*	-	1	
Other medication use			
Anti-convulsant	-	3	
Muscle relaxant	-	1	
Analgesic	-	1	
Anti-depressant	2	2	
Antibiotic	1	-	
Thyroid medication	2	-	
Bladder medication	-	2	
Anti-asthmatic	1	-	
Contraceptive	2	3	
Vitamin D	-	3	
Folic acid	-	2	
Anti-allergy	-	2	
Smoker, *n*	-	4	
Cannabis use, *n*	-	2	

Data are presented as median (interquartile range) or *n.* EDSS: Expanded Disability Status Scale; HC: Healthy control cases; MS: Multiple sclerosis; MSQOL-54: MS Quality of Life-54; QIDS-SR_16_: self-rated 16-item Quick Inventory of Depressive Symptomatology. n/a: not applicable. EDSS were available for 13 pwMS. MSQOL-54 questionnaire data are from 23 HCs and 20 pwMS. QIDS-SR_16_ questionnaire data are from 23 HCs and 17 pwMS. *** *p* < 0.001 compared to HCs.

**Table 2:** Constitutive expression of *TLR3* and *TLR4* mRNA in PBMCs from HCs and pwMS.

Target Gene	HC	MS	*p* Value
TLR3 median delta Ct *	20.9 (20.2–21.8)	21.3 (19.4–21.5)	0.9999
TLR4 median delta Ct	14.8 (14.2–17.9)	14.8 (14.0–17.1)	0.3095

The table includes median delta Ct values (interquartile range) for *TLR3* and *TLR4* mRNA in untreated PBMCs. HC: Healthy control; MS: Multiple sclerosis. Values are available for 24 HC cases and 16–17 pwMS. Statistical analysis: Mann-Whitney test. * delta Ct = Ct of target gene–Ct of housekeeping gene.

**Table 3:** Production of CXCL10 and IFN-β in the absence and presence of poly(I:C) in PBMCs from HC cases and pwMS.

Cytokine/Chemokine	Basal	Treatment with Poly(I:C)	Mean Difference after Treatment (Fold Change)
CXCL10 (pg/mL)			
HC	74.6 ± 13.3	587.6 ± 94.1 ***	513.0 (7.9)
MS	28.0 ± 7.5	289.6 ± 71.6 ^#^	261.6 (10.3)
IFN-β (pg/mL)			
HC	0.68 ± 0.22	2.46 ± 0.39 *	1.78 (3.6)
MS	0.72 ± 0.18	3.39 ± 0.68 ^+++^	2.67 (4.7)

The table includes mean CXCL10 and IFN-β values in culture media ± SEM. * *p* < 0.05, *** *p* < 0.001 versus basal CXCL10 and IFN-β production in PBMCs from HC cases. ^#^ *p* < 0.05 versus CXCL10 production in poly(I:C)-treated PBMCs from HC cases. ^+++^ *p* < 0.001 versus basal IFN-β production in PBMCs from MS cases. HC: Healthy control; MS: Multiple sclerosis. Values are available for 20–24 HC cases and 14–15 pwMS. Statistical analysis: two-way ANOVA.

**Table 4:** Production of TNF-α in the absence and presence of LPS in PBMCs from HC cases and pwMS.

Cytokine	Basal	Treatment with LPS	Mean Difference after Treatment (Fold Change)
TNF-α (pg/mL)			
HC	69.3 ± 19.8	2398 ± 208.2 ***	2328.7 (34.6)
MS	504.4 ± 113.3	2377 ± 177.6 ^+++^	1872.6 (4.7)

The table includes mean TNF-α values in culture media ± SEM. *** *p* < 0.001 versus basal production in PBMCs from HC cases. ^+++^ *p* < 0.001 versus basal production in PBMCs from MS cases. HC: Healthy control; MS: Multiple sclerosis. Values are available for 22 HC cases and 19 pwMS. Statistical analysis: two-way ANOVA.

**Table 5:** Constitutive expression of *CNR1*, *CNR2*, *FAAH,* and *MGLL* mRNA in PBMCs from HCs and pwMS.

Target Gene	HC	MS	*p* Value
CNR1 average delta Ct *	20.8 ± 0.2	20.3 ± 0.9	0.4635
CNR2 average delta Ct	17.1 ± 0.2	16.6 ± 0.4	0.1867
FAAH average delta Ct	19.1 ± 0.2	18.7 ± 0.4	0.2802
MGLL average delta Ct	13.4 ± 0.3	13.1 ± 0.6	0.6103

The table includes mean delta Ct values ± SEM for *CNR1*, *CNR2*, *FAAH* and *MGLL* in untreated PBMCs. CNR1: cannabinoid receptor 1; CNR2: cannabinoid receptor 2; FAAH: fatty acid amide hydrolase; HC: Healthy control; MGLL: monoacylglycerol lipase; MS: Multiple sclerosis. Values are available for 19–24 HC cases and 9–17 pwMS. Statistical analysis: Unpaired *t*-test. * delta Ct = Ct of target gene–Ct of housekeeping gene.

## Data Availability

Data are contained within the article and the Supplementary Materials.

## Supplementary Materials

The following supporting information can be downloaded at: https://www.mdpi.com/article/10.3390/molecules27061763/s1, Table S1. Detection rate and value (pg/mL) of plasma CRP. Table S2. Comparison of blood parameters in HC and pwMS.

## Sample Availability

Samples are not available from the authors.
