# Supplementary material for: Botanically-Derived Δ9-Tetrahydrocannabinol and Cannabidiol, and Their 1:1 Combination, Modulate Toll-like Receptor 3 and 4 Signalling in Immune Cells from People with Multiple Sclerosis
Source: Molecules. 2022 Mar 8;27(6):1763. doi: 10.3390/molecules27061763 (PMC8951523; doi:10.3390/molecules27061763)
Supplement: Supplementary file 1 [file molecules-27-01763-s001.zip › molecules-1586529-supplementary.pdf]

Supplementary Table S1. Detection rate and value (pg/ml) of plasma CRP.

Category	HC		HC		
	**Detection rate**	**Median (range)**	**Median (range)**	**Median (range)**	***p* value**
CRP	25/25	1734 (1383-1891)	20/20	1703 (1682-1911)	0.5734

The table includes median protein values (pg/ml) of CRP that could be detected. Detection rates are also shown. Plasma was available for 25 HC cases and 20 pwMS. Data are presented as median (interquartile range). Statistical analysis: Mann-Whitney test. HC: Healthy control; MS: Multiple sclerosis.

Supplementary Table S2. Comparison of blood parameters in HC and pwMS.

Parameter	HC	MS	*p* value
WBC ($X10^3/\mu L$)	6.1 ± 0.3	5.6 ± 0.5	0.3564
RBC ($X10^6/\mu L$)	4.8 ± 0.2	4.8 ± 0.2	0.9970
HGB (g/dL)	14.8 ± 0.4	15.2 ± 0.5	0.4811
PLT ($X10^3/\mu L$)	255.1 ± 16.1	243.7 ± 18.2	0.6384
HCT (%)	40.9 ± 1.2	41.5 ± 1.4	0.7347
MCV (fL)	84.7 ± 0.7	85.9 ± 0.8	0.2510
MCH (pg)	30.1 ± 0.4	31.2 ± 0.5	0.0720
MCHC (g/dL)	35.4 ± 0.3	36.4 ± 0.3	0.0251*

Data are presented as mean ± SEM. HC: Healthy control; HCT: haematocrit; HGB: haemoglobin; MCH: mean corpuscular haemoglobin; MCHC: mean corpuscular haemoglobin concentration; MCV: mean corpuscular volume; MS: Multiple sclerosis; PLT: platelet number; RBC: red blood cell number; WBC: white blood cell number. Statistical analysis: Student's *t*-test. *$p<0.05$ versus basal MCHC in HC cases. Sysmex readings were available for 24 HC cases and 20 pwMS.
